# Preventive Antibiotic Use and Complications After Endoscopic Retrograde Cholangiopancreatography in Patients Hospitalized for Primary Sclerosing Cholangitis

**DOI:** 10.7759/cureus.64429

**Published:** 2024-07-12

**Authors:** Fidelis E Uwumiro, Solomon O Anighoro, Michael M Bojerenu, Nsikan N Akpabio, Samuel U Asogwa, Victory Okpujie, Hillary Alemenzohu, Osarumwense D Ufuah, Miracle C Okoro, Ihunanya M Kanu, Tosin Ayantoyinbo, Ridwan A Lawal

**Affiliations:** 1 Internal Medicine, Our Lady of Apostles Hospital, Akwanga, NGA; 2 General Medicine, St. Helens and Knowsley Teaching Hospitals NHS Trust, Prescot, GBR; 3 Internal Medicine, St. Barnabas Hospital SBH Heath System, New York, USA; 4 Medicine and Surgery, Bingham University Teaching Hospital, Jos, NGA; 5 Internal Medicine, London North West University Healthcare NHS Trust, Harrow, GBR; 6 Internal Medicine, Central Hospital Benin, Benin City, NGA; 7 Internal Medicine, College of Medicine, University of Ibadan, Ibadan, NGA; 8 Family Medicine, All Saints University College of Medicine, Kingstown, VCT; 9 Internal Medicine, Imo State University College of Medicine, Owerri, NGA; 10 Internal Medicine, Jackson State University, Jackson, USA; 11 Internal Medicine, Obafemi Awolowo College of Health Sciences, Olabisi Onabanjo University, Ogun State, NGA; 12 Internal Medicine, College of Medicine, University of Lagos, Lagos, NGA

**Keywords:** acute biliary pancreatitis, acute cholangitis, primary sclerosing cholangitis, septicemia, endoscopic retrograde cholangiopancreatography, antibiotic prophylaxis

## Abstract

Background: The American Society for Gastrointestinal Endoscopy recommends prophylactic antibiotics before endoscopic retrograde cholangiopancreatography (ERCP) in primary sclerosing cholangitis (PSC). We assessed the impact of this approach on the incidence of post-ERCP outcomes using nationwide data.

Methods: Using 2015-2021 Nationwide Inpatient Sample data and relevant ICD-10 codes, we analyzed adult hospitalizations for PSC who underwent ERCP, with and without antibiotic prophylaxis. Hierarchical multivariate logistic regression analysis was used to assess the association between prophylactic antibiotic use and post-ERCP complications including sepsis, acute cholangitis, and acute pancreatitis.

Results: We analyzed 32,972 hospitalizations for PSC involving ERCP, with 12,891 admissions (39.1%) receiving antibiotics before ERCP (cases) and 20,081 (60.9%) serving as controls. Cases were older than controls (mean age: 64.2 ± 8.6 vs. 61.3 ± 6.1 years; P = 0.020). Compared with controls, hospitalizations with antibiotic prophylaxis had a higher male population (7,541 (58.5%) vs. 11,265 (56.1%); P < 0.001) and higher comorbidity burden (Charlson comorbidity index score of ≥2: 5,867 (45.5%) of cases vs. 8,996 (44.8%) of controls; P = 0.01). Incidence of post-ERCP septicemia was 19.1% (6,275) with 2,935 incidences (22.8%) among cases compared with 3,340 (16.6%) among controls. Antibiotic prophylaxis did not significantly improve the odds of septicemia (aOR: 0.85; 95% CI: 0.77 - 1.09; P = 0.179). Approximately 2,271 (6.9%) cases of acute cholangitis and 5,625 (17.1%) cases of acute post-ERCP pancreatitis were recorded. After adjustments for multiple variables, no significant difference was observed in the odds of cholangitis (aOR: 0.87; 95% CI: 0.98 - 1.45; P = 0.08). However, antibiotic prophylaxis was correlated with a statistically significant reduction in the odds ratio of acute post-ERCP pancreatitis (aOR: 0.61; 95% CI: 0.57 - 0.66; P < 0.001).

Conclusion: The use of antibiotic prophylaxis in hospitalizations with PSC was correlated with a significant reduction in the odds of post-ERCP pancreatitis. Antibiotic prophylaxis did not improve the odds of post-ERCP sepsis or cholangitis. Prophylactic use of antibiotics should be individualized, considering both their anti-infective benefits and potential impact on the biochemical markers of liver disease.

## Introduction

Endoscopic retrograde cholangiopancreatography (ERCP), with a specificity approaching 96%, is recommended by the European Society of Gastrointestinal Endoscopy (ESGE) and the European Association for the Study of the Liver (EASL) when magnetic resonance cholangiography (MRC) and liver biopsy results are inconclusive or contraindicated but clinical suspicion of PSC still exists, underlining the importance of considering benefits versus risks [1]. ERCP should follow MRC in treating significant strictures, with advanced techniques like papillotomy/sphincterotomy used after challenging cannulations. For patients with PSC exhibiting advanced disease, ERCP plays an essential role in the diagnosis and staging of cholangiocarcinoma [2]. Prophylactic antibiotics are strongly recommended before performing ERCP to prevent infections, with the Enterobacteriaceae family, especially Escherichia coli and Enterococcus faecium, implicated as the predominant pathogens in post-ERCP infections [2-8].

Despite evidence suggesting no significant impact on infectious outcomes following ERCP, the American Society for Gastrointestinal Endoscopy and the British Society of Gastroenterology continue to endorse antibiotic prophylaxis for patients with primary sclerosing cholangitis (PSC) [9-12]. We examined the risk of post-ERCP infections in hospitalized PSC patients with and without antibiotic prophylaxis.

## Materials and methods

Data source

The National Inpatient Sample (NIS) is part of a group of databases and software tools developed for the Healthcare Cost and Utilization Project (HCUP). The NIS is the largest all-payer inpatient care database in the U.S., containing unweighted data on more than seven million hospital stays every year since 1988. Beginning with the 2012 data year, the NIS was redesigned to approximate a 20% stratified sample of all discharges from US community hospitals, excluding rehabilitation and long-term acute care hospitals. Weights are provided to estimate national case volumes from the 20% sample. When weighted, each year of NIS data represents more than 97% of the U.S. population, 96% of discharges across community hospitals, and up to 35 million hospitalizations across 47 states, including the District of Columbia. Its large sample size is ideal for developing national and regional estimates and enables analyses of rare conditions, uncommon treatments, and special patient populations [13].

The NIS provides records of patient demographics, comorbidities, primary and secondary procedures performed, insurance coverage, associated hospital costs, and in-hospital outcomes [14]. It captures the main reason for hospitalization as the primary diagnosis and records additional diagnoses as secondary diagnoses. All diagnoses and procedures within the NIS database from October 1, 2015, to date were recorded using the International Classification of Diseases, Tenth Revision, Clinical Modification, and Procedure Coding System (ICD-10 CM/PCS). In addition, the NIS records the time from admission to the performance of a specific procedure, allowing the analysis of time relationships between procedures. Each entry in the NIS details up to 40 diagnoses and 25 procedures for each hospitalization. Every hospitalization is recorded as a distinct, deidentified observation, with one primary diagnosis and up to 39 secondary diagnoses. The primary diagnosis is the principal diagnosis associated with hospitalization (essentially the reason for the hospitalization), whereas secondary diagnoses include all other diagnoses (comorbidities and acute in-hospital complications).

The U.S. Agency for Healthcare Research and Quality (AHRQ) designs and regulates the use of NIS, a publicly accessible database created through the HCUP. NIS is completely deidentified and adheres to the highest standard in patient health data privacy and confidentiality. The use of the NIS, which is classified as a limited dataset, for the index study is exempt from IRB review [15,16].

Study selection and exclusion criteria

**Figure 1:**
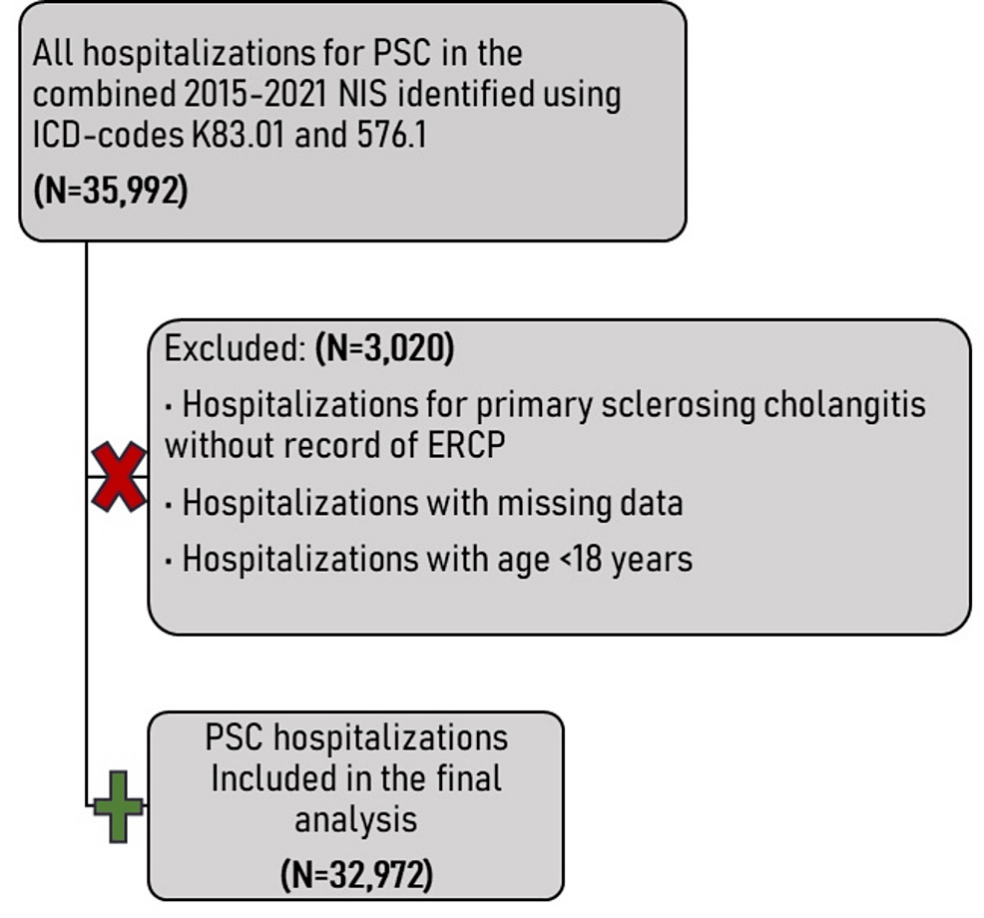
Derivation of the study cohort PSC, primary sclerosing cholangitis; NIS, nationwide inpatient sample; ICD, international classification of diseases; ERCP, endoscopic retrograde cholangiopancreatography

All adult hospitalizations with a primary diagnosis of PSC between January 1, 2015, and December 31, 2021, were identified in the NIS using the International Classification of Diseases, Tenth Revision, Clinical Modification (ICD-10-CM/PCS) code K83.01 for hospitalizations from October 2015 to December 2021. Admissions for PSC from January 1 to September 30, 2015, were identified using ICD-9 code 576.1. Hospitalizations with ERCP were identified using the ICD-10 procedure codes (Table 3 and Table 4 of Appendix). The derivation of our study cohort is summarized in Figure 1.

Acute post-ERCP complications, such as post-ERCP septicemia, acute cholangitis, and acute pancreatitis, were identified using relevant ICD-10 diagnostic codes (Appendix) from secondary diagnostic variables. Antibiotic prophylaxis was defined by the presence of the ICD-10 procedure code for the introduction of anti-infectives into the peripheral vein (ICD-9: 99.21; ICD-10: 3E03029) and the administration of anti-infectives before ERCP (defined as time-to-introduction of anti-infectives < time-to-ERCP). Hospitalizations were excluded if they involved missing data, were aged under 18 years old, or lacked ICD-9 or ICD-10 procedure codes for ERCP. In addition, because the NIS records time-to-procedures in days, we excluded ERCPs performed on the day of admission because of the difficulty of establishing a time relationship between ERCP and antibiotic prophylaxis.

The study included other variables, including demographic information and resource utilization. These variables included age, gender, race, median annual income, hospital region, bed size, teaching status, day of admission (weekend vs. weekday), and type of admission (elective, nonelective, or transfers from other facilities). The comorbidity burden was assessed using the Charlson-Deyo Comorbidity Index (CCI) [17]. The Deyo-CCI represents an adaptation of the original CCI refined to align with modern diagnostic codes [18]. It includes multiple comorbidities including myocardial infarction, congestive heart failure, peripheral vascular disease, cerebrovascular disease, dementia, chronic pulmonary disease, connective tissue disease, peptic ulcer disease, mild and moderate/severe liver disease, diabetes with and without complications, hemiplegia or paraplegia, renal disease, any malignancy including leukemia and lymphoma, metastatic solid tumor, and AIDS/HIV. This streamlined and widely accessible tool was selected for use in our study because of its simplicity and effectiveness in evaluating the comorbidity burden. We assessed baseline illness severity and mortality risk at admission using the All Patient Refined-Diagnosis Related Groups (APR-DRG) system, which calculates severity and mortality risk scores for each hospitalization. These scores were derived from diagnostic codes, factoring in primary and secondary diagnoses, age, and preexisting conditions, but excluding codes for complications developed during the hospital stay. The APR-DRG system categorizes each hospitalization into one of four distinct levels of functional loss (LOF) and likelihood of dying (LOD): minor, moderate, major, and extreme, facilitating the assessment and adjustment of illness severity and mortality risk in our study. A previous study assessed the validity of the APR-DRG system [19].

The primary endpoint of the study was the incidence of post-ERCP septicemia. Secondary outcomes included the incidence of acute cholangitis and post-ERCP pancreatitis with and without antibiotic prophylaxis. Septicemia was defined as the presence of ICD-10 codes for streptococcal sepsis (A40.X), other sepsis (A41.X), severe sepsis (R65.2), post-ERCP bacteremia (R78.81), pneumonia (J13, J15.9, J18.0, J18.8, J18.9, J85.2), peritonitis (K65.9), and cholecystitis (K81.0, K83.0). The reliability of ICD-10 coding for sepsis in administrative databases has been explored in previous studies [20-22].

Statistical analyses

The index study’s analyses were based on weighted NIS data, accounting for clustering (HOSP_NIS), weighting (DISCWT), and stratification (NIS_STRATUM) for nationwide representation of the inpatient PSC population. We used Pearson’s χ2 test to compare sociodemographic variables between PSC hospitalizations with and without antibiotic prophylaxis. The Shapiro-Wilk test was used to determine the normality of the continuous data distribution. Data with normal distributions are presented as means and SD, whereas those with non-normal distributions are presented using medians with interquartile ranges (IQR). Using stepwise multivariate logistic regression analysis, we determined the probabilities of primary and secondary outcomes. To mitigate the interference of multiple admissions of the same patient on independence of hospitalizations included in the study cohort, we employed hierarchical multivariate logistic regression analysis, which accounted for the nested structure of the data. Variables with P-values below 0.1 in the univariate analysis, such as history of liver transplantation, bile duct perforation, hemorrhage, advanced age (≥ 85 years), female gender, need for sphincterotomy, higher Charlson comorbidity index, physical frailty, multiple procedures, July effect, and length of hospital stay identified from existing literature, were entered into the multivariable logistic regression model. The post hoc variance inflation factor (VIF) method was used to evaluate multicollinearity, with a threshold of five [23]. Covariates showing significant collinearity were removed to maintain a stable multivariate regression analysis [24]. The Johns Hopkins Adjusted Clinical Group frailty clusters were used to define frailty [25,26]. The regression results are given as adjusted odds ratios with accompanying 95% CI; analysis was conducted using Stata version 18MP (StataCorp LLC, College Station, TX).

## Results

Sociodemographic characteristics and resource use 

We analyzed 32,972 hospitalizations with a primary diagnosis of PSC during which an ERCP was performed (see Figure 1). Among these, 12,891 (39.1%) received antibiotics before the procedure (study group or cases), while 20,081 hospitalizations (60.9%) did not (controls). Cases were older than controls (mean age: 64.2 ± 8.6 vs. 61.3 ± 6.1 years; P = 0.020). Compared with controls, hospitalizations with antibiotic prophylaxis had a higher male population (7,541 (58.5%) vs. 11,265 (56.1%); P < 0.001) and higher comorbidity burden (Charlson comorbidity index score of ≥2: 5,867 (45.5%) of cases vs. 8,996 (44.8%) of controls; P = 0.01). Both groups were mostly composed of white Americans and Hispanics. Most hospitalizations in both subgroups were nonelective (12,272 (95.2%) vs. 18,575 (92.5% of controls)). In addition, 7.1% (915) of the study group were uninsured, compared to 4.9% (984 hospitalizations) of controls. Insured hospitalizations in both subgroups had either Medicare (6,935 (53.8%) vs. 10,201 (50.8%)) or Medicaid (1,998 (15.5%) vs. 2,972 (14.8%)) and were admitted to medium or large urban teaching hospitals, primarily located in the southern and western regions of the United States. A total of $8.7 billion was spent on hospital costs for hospitalizations included in this study. There was no significant difference in the mean cost of healthcare financing between the two study subgroups (P = 0.175; Table 1).

**Table 1:** Sociodemographic characteristics and resource utilization for PSC hospitalizations with ERCP dichotomized by antibiotic prophylaxis Categorical data is presented as absolute counts (N) with percentages (%). Continuous data is presented as means ± standard deviation. ^b^Sundararajan's adaptation of the modified CCI HMO, health maintenance organization; SNF, skilled nursing facility; ICF, intermediate care facility; ERCP, endoscopic retrograde cholangiopancreatography; LOF, loss of function; LOD, likelihood of dying; CCI, Charlson-Deyo comorbidity index

Variable	ERCP With Prophylactic Antibiotics (N = 12,891)	ERCP Without Prophylactic Antibiotics (N = 20,081)	P
Mean age ± SD, years	64.2 ± 8.6	61.3 ± 6.1	0.020
Women	5,350 (41.5)	8,816 (43.9)	<0.001
Men	7,541 (58.5)	11,265 (56.1)	<0.001
Mean hospital charge, US$	81,458 ± 2,844	86,619 ± 1,968	0.464
Aggregate hospital charge, billion $US	3.6	5.1	0.175
Elective admission	619 (4.8)	1,506 (7.5)	<0.001
Emergency admission	12,272 (95.2)	18,575 (92.5)	0.658
Mean length of stay, days ± SD	11.3 ± 6.4	15.5 ± 3.1	0.219
APR-DRG severity of the illness			0.322
Minor LOF	0 (0.0)	301 (1.5)	-
Moderate LOF	5,956 (46.2)	9,599 (47.8)	
Major LOF	5,453 (42.3)	8,394 (41.8)	
Extreme LOF	1482 (11.5)	1,807 (9.0)	
APR-DRG risk of mortality			0.377
Minor LOD	4,654 (36.1)	6,004 (29.9)	-
Moderate LOD	4,460 (34.6)	9,298 (46.3)	
Major LOD	2,784 (21.6)	3,595 (17.9)	
Extreme LOD	993 (7.7)	1,205 (6.0)	
Discharge disposition			<0.001
Died in the hospital	77 (0.6)	482 (2.4)	-
Routine home discharge	10,596 (82.2)	14,719 (73.3)	
Transfer to a short-term hospital	155 (1.2)	361 (1.8)	
Transfer to other SNF, home health care, and ICF	1,934 (15)	4,578 (22.8)	
Against medical advice	129 (1.0)	120 (0.6)	
Discharge quarter			<0.001
First quarter	3,261 (25.3)	4,920 (24.5)	-
Second quarter	3,042 (23.6)	4,920 (24.5)	
Third quarter	3,403 (26.4)	5,201 (25.9)	
Fourth quarter	3,184 (24.7)	5,040 (25.1)	
Race			0.01
White American	8,418 (65.3)	13,474 (67.1)	-
Black	1,341 (10.4)	1,827 (9.1)	
Hispanic	1,392 (10.8)	3,113 (15.5)	
Asian/Pacific islander	1,031 (8.0)	823 (4.1)	
Native American	64 (0.5)	161 (0.8)	
Others	645 (5.0)	683 (3.4)	
Weekend admission	3713 (28.8)	4,799 (23.9)	0.010
Combined Charlson comorbidity index^b^			0.010
0	4,022 (31.2)	7,048 (35.1)	-
1	3,004 (23.3)	4,036 (20.1)	
2	2,010 (15.6)	2,751 (13.7)	
≥3	3,854 (29.9)	6,245 (31.1)	
Median annual income quartiles, US$			0.396
1-43,999	3,042 (23.6)	5,422 (27.0)	-
44,000-55,999	2,681 (20.8)	5,362 (26.7)	
56,000-73,999	3,339 (25.9)	4,960 (24.7)	
≥74,000	3,700 (28.7)	4,317 (21.5)	
Insurance status			0.06
Medicare	6,935 (53.8)	10,201 (50.8)	-
Medicaid	1,998 (15.5)	2,972 (14.8)	
Private, including HMO	3,042 (23.6)	5,904 (29.4)	
Self-pay	915 (7.1)	984 (4.9)	
Hospital location			0.03
Rural	322 (2.5)	623 (3.1)	-
Urban-non-teaching	1,173 (9.1)	3,896 (19.4)	
Urban teaching hospital	11,395 (88.4)	15,543 (77.4)	
Hospital region			0.201
Northeast	1,650 (12.8)	3,695 (18.4)	-
Midwest	3,622 (28.1)	4,457 (22.2)	
South	4,293 (33.3)	7,189 (35.8)	
West	3,326 (25.8)	4,739 (23.6)	
Hospital bed size			0.478
Small	1,921 (14.9)	3,052 (15.2)	-
Medium	2,952 (22.9)	5,442 (27.1)	
Large	8,018 (62.2)	11,587 (57.7)	
Liver cirrhosis	5,002 (38.8)	8,093 (40.3)	0.076
Cholangiocarcinoma	670 (5.2)	1,104 (5.5)	0.274
Ulcerative colitis	4,151 (32.2)	6,085 (30.3)	0.009
Crohn's disease	1,818 (14.1)	2,651 (13.2)	0.043

Antibiotic prophylaxis and post-ERCP sepsis

The study recorded a total of 6,275 cases of post-ERCP septicemia (19%). Among these, 3,340 (53.2%) occurred among controls, whereas 2,935 (46.7%) were recorded among the study group. Approximately 16.6% of the controls developed septicemia, compared with 22.8% of the study group. After adjustments in a multivariate regression analysis, antibiotic prophylaxis did not significantly affect the odds of post-ERCP septicemia (adjusted odds ratio (aOR): 0.85; 95% CI: 0.77-1.09; P = 0.179; Table 2).

**Table 2:** Multivariate regression for post-ERCP septicemia ^a^Significant at values <0.05 ^b^Sundararajan's adaptation of the modified CCI ^c^Performance of five or more procedures in one admission ^d^Defined as admission during the early academic months for resident physicians (July-September) aOR, adjusted odds ratio; CI: confidence interval; ERCP, endoscopic retrograde cholangiopancreatography; CCI, Charlson-Deyo Comorbidity Index

Predictor variable	aOR (95% CI)	P^a^
Antibiotic prophylaxis	0.85 (0.77 - 1.09)	0.179
History of liver transplantation	1.07 (1.06 - 1.14)	0.040
Bile duct perforation	1.85 (1.53 - 1.98)	<0.001
Severe hemorrhage	1.68 (1.31 - 1.86)	<0.001
Elderly (≥85 years)	1.99 (1.76 - 2.08)	<0.001
Female vs. male	1.01 (0.96 - 1.06)	0.265
Post-ERCP cholangitis	1.91 (1.71 - 1.16)	0.030
Need for a sphincterotomy	0.98 (0.95 - 1.21)	0.470
Post-ERCP pancreatitis	1.46 (1.29 - 1.65)	<0.001
Ulcerative colitis	1.17 (0.98 - 3.17)	0.245
Crohn's disease	0.79 (0.65 - 1.91)	0.482
Cholangiocarcinoma	2.13 (0.91 - 4.18)	0.349
Liver cirrhosis	3.33 (1.84 - 4.58)	0.029
Admission to urban teaching hospitals vs. rural nonteaching hospital	0.17 (0.09 - 0.25)	<0.001
Admission to large hospitals vs. small hospitals	1.58 (1.39 - 2.25)	<0.001
CCI≥2^b^	4.47 (3.39 - 5.26)	<0.001
Physical frailty	2.11 (1.96 - 3.45)	0.040
Multiple procedures^c^	3.12 (2.75 - 3.98)	<0.001
July effect^d^	1.48 (1.20 - 1.57)	<0.001
Weekend vs. weekday admission	0.97 (0.84 - 1.25)	0.478
Elective vs. nonelective admission	1.87 (1.05 - 2.19)	0.030
Length of hospital stay >3 vs. ≤3 days	2.66 (1.80 - 3.06)	0.010

Significant predictors of post-ERCP sepsis identified in the study included a history of liver transplantation, coexisting liver cirrhosis, ERCP complications such as bile duct perforation, severe hemorrhage, post-ERCP cholangitis, and post-ERCP-complicated pancreatitis. Other predictors included admission at large or urban teaching hospitals, undergoing ≥5 procedures during the same hospitalization, admission between July and September, and hospitalization lasting ≥3 days.

Post-ERCP cholangitis and pancreatitis

Approximately 2,271 (6.9%) cases of acute cholangitis and 5,625 (17.1%) cases of acute post-ERCP pancreatitis were recorded. Most cases of post-ERCP cholangitis and pancreatitis (1,165 (51.3%) and 3,728 (66.3%), respectively) were observed among controls. In contrast, the study cohort accounted for 1,106 cases of cholangitis (48.7%) and 1,895 (33.7%) cases of post-ERCP pancreatitis. Comparing cases and controls, the unadjusted odds ratio of post-ERCP cholangitis was 1.62 (95% CI: 1.51 - 1.76; P < 0.001). After adjustments for all patient- and hospital-level covariates, no significant difference was observed in the odds ratio of post-ERCP cholangitis (aOR: 0.87; 95% CI: 0.98 - 1.45; P = 0.08). The unadjusted odds ratio of post-ERCP pancreatitis was 0.79 (95% CI: 0.74 - 0.85; P < 0.001), indicating a lower likelihood of pancreatitis in the antibiotic prophylaxis group. After adjustments for all patient- and hospital-level covariates, antibiotic prophylaxis was correlated with a statistically significant reduction in the likelihood of acute post-ERCP pancreatitis (aOR: 0.61; 95% CI: 0.57 - 0.66; P < 0.001).

## Discussion

Our study aimed to explore the efficacy of routine prophylactic antibiotics in preventing infectious post-ERCP complications among patients with PSC. The findings of the index study showed that antibiotic prophylaxis did not significantly reduce the odds of post-ERCP sepsis. This finding is consistent with those of prior studies that have questioned the routine administration of prophylactic antibiotics in ERCP procedures [27,28]. The risk of sepsis after invasive procedures is driven by other factors, such as patient characteristics, procedural complications, or healthcare-associated infections, rather than the lack of antibiotic prophylaxis. Specifically, antibiotic resistance could diminish the effectiveness of prophylaxis, especially because they are empirically administered and antibiotic selection may not be optimal for the pathogens implicated. PSC often leads to complex biliary anatomy, increasing infection risks regardless of antibiotic use. Advanced disease stages, inadequate biliary drainage, technical shortcomings in ERCP, or breaches in procedural sterility could also contribute to high infection risks. In addition, variations in patient immune status and unique microbial flora in the biliary system may not respond well to standard antibiotics, undermining the protective effects of prophylaxis.

Our study also identified several predictors of post-ERCP sepsis, including a history of liver transplantation and ERCP complications such as bile duct perforation, hemorrhage, post-ERCP cholangitis, and post-ERCP pancreatitis. These findings underscore the complex nature of septicemia in the post-ERCP setting. It is plausible that these risk factors play a more significant role in determining the occurrence of sepsis than antibiotic prophylaxis. For instance, the presence of ERCP complications can lead to the introduction of bacteria into the biliary system, increasing the risk of subsequent septicemia. Similarly, patients with a history of liver transplantation may have compromised immune systems or other underlying factors that make them more susceptible to infections [29].

The lack of a significant difference in the odds of post-ERCP cholangitis between the study groups indicates that antibiotic prophylaxis may not be effective in preventing this specific complication. It is possible that the mechanisms underlying post-ERCP cholangitis are multifactorial and are not solely related to the presence or absence of antibiotic prophylaxis. Other factors, such as procedural technique, the presence of preexisting biliary obstruction or infection, and the host immune response, may contribute to the development of cholangitis [30]. Future research should investigate these factors to better understand the pathogenesis of post-ERCP cholangitis and develop targeted preventive strategies. Our study found a statistically significant reduction in the likelihood of acute post-ERCP pancreatitis with antibiotic prophylaxis. This result aligns with prior studies suggesting a potential benefit of prophylactic antibiotics in preventing pancreatitis following ERCP [31,32]. One possible explanation for this finding is that antibiotic prophylaxis effectively reduces the bacterial load in the pancreatic duct and decreases the risk of infection-induced pancreatitis. However, the exact mechanisms underlying this observed benefit and the incidence of other noninfectious pancreatitis require further exploration. Future studies should examine the specific pathogens associated with post-ERCP pancreatitis and evaluate the effectiveness of different antibiotic regimens to provide more targeted recommendations.

It is worth noting that the cost of healthcare financing did not significantly differ between the study groups. This finding indicates that the implementation of antibiotic prophylaxis did not result in substantial cost differences, suggesting that the potential benefits of preventing post-ERCP pancreatitis may outweigh the associated healthcare costs. However, further economic evaluations, considering long-term outcomes and cost-effectiveness, are needed to inform decision-making regarding the use of prophylactic antibiotics in this patient population.

Beyond the debate on the efficacy of prophylactic antibiotics in preventing infections post-ERCP complications, antibiotic use in PSC has also been linked with a statistically significant reduction in alkaline phosphatase, Mayo PSC risk score, and total serum bilirubin by up to 30% in recent studies [33-37]. Thus, the decision to use antibiotics in patients with PSC needs to be individualized, considering both their potential to prevent complications post-ERCP and their impact on biochemical markers of liver disease. Given the evidence that antibiotics can significantly reduce alkaline phosphatase levels, the Mayo PSC risk score, and total serum bilirubin, which are key indicators of disease activity and progression in PSC, the use of antibiotics may be beneficial beyond just prophylactic use. Prophylaxis against spontaneous bacterial peritonitis (SBP) in patients with cirrhotic ascites is a widely accepted practice in hepatology [38]. This is especially crucial for patients who have previously recovered from an episode of SBP (secondary prophylaxis). Antibiotic prophylaxis reduces the risk of sepsis in patients with cirrhosis undergoing ERCP by decreasing the bacterial load in the biliary system and gut, thereby preventing bacterial translocation into the bloodstream. It helps minimize the risk of infections such as cholangitis and pancreatitis, which can arise as complications of the procedure. Additionally, antibiotics bolster the compromised immune defense of patients with cirrhosis and protect against the exacerbation of pre-existing infections like SBP. Collectively, these measures significantly lower the likelihood of sepsis in these vulnerable patients. Cirrhotic patients are at greater risk of sepsis after ERCP due to several factors: immune dysfunction, increased bacterial translocation from the gastrointestinal tract, and impaired hepatic clearance of bacteria and endotoxins. These patients often present with biliary obstructions that ERCP can manage but increase the risk of infection. Additionally, complications such as ascites and SBP, coagulopathy, and procedural complications like pancreatitis and cholangitis further increase susceptibility. Together, these factors increase the risk of sepsis in patients with cirrhosis who undergo ERCP.

Limitations

Our study, while offering valuable discoveries, includes some drawbacks. The reliance on administrative data and retrospective design in research can introduce inherent biases and limitations when capturing clinical details and outcomes. The accuracy and completeness of case identification using ICD codes could be restricted. The study's lack of data on antibiotic type, dosage, and duration may have impacted the assessment of antibiotic prophylaxis effectiveness. Because of the nested data structure and the lack of direct patient identifiers, patients with the same diagnosis readmitted during the study period may appear multiple times in the study cohort. To mitigate this problem, we employed hierarchical multivariate logistic regression, also known as mixed-effects or multilevel logistic regression. It mitigates the problem of multiple admissions of the same patient by including random effects for patients, capturing within-patient correlation, and adjusting for patient-specific variability. This method acknowledges the nested data structure, preventing the underestimation of standard errors and reducing the risk of overfitting by treating multiple admissions from the same patient as correlated rather than independent events. This approach offers flexibility in model specification, allowing for comprehensive modeling of different hierarchy levels, and improves the generalizability of the results by providing more accurate estimates that distinguish between individual patient differences and other factors. Because the NIS database does not provide specific causes of death or indications for each procedure, we could not distinguish between patients who received antibiotics as prophylaxis for ERCP and those who received antibiotics to treat other pre-procedure infections that may have contributed to hepatic decompensation. The findings of the index study are drawn from analyses of hospitalization data and may not be generalizable to ERCPs performed as day cases in cohorts of PSC patients with milder disease. Given the limited data on specific reasons for hospitalizations, it would be useful to determine if other medical conditions contributed to the hospitalizations and whether these conditions prolonged the hospital stays, thereby increasing the likelihood of post-procedure complications, important areas for future research.

## Conclusions

Antibiotic prophylaxis did not correlate with reduced odds of post-ERCP sepsis or cholangitis. However, antimicrobial prophylaxis dramatically lowered the risk of acute pancreatitis following ERCP. Antibiotic prophylaxis should be used based on an individual patient risk assessment, with the goal of balancing anti-infective effects with the potential effect of lowering liver disease biomarkers. Identifying specific patient subgroups that may benefit the most from antibiotic prophylaxis, as well as clarifying the processes underlying the reported improvements in pancreatitis prevention, will considerably improve our understanding, contribute to ongoing efforts to minimize antibiotic resistance, and serve as a focus for future studies. 

## Appendices

**Table 3:** Methodological checklist for studies performed using the nationwide inpatient sample database

Section A: Research Design
1. Does the study consider that it can only detect disease conditions, procedures, and diagnostic tests in hospital settings? Yes
2. Does the study acknowledge that it includes encounters, not individual patients? Yes. We have consistently used “hospitalizations” rather than “patients” wherever it is applicable.
Section B. Data Interpretation
3. Does the study attempt to identify disease conditions or procedures of interest using administrative codes or their combinations that have been previously validated? Yes. We have identified all the disease conditions and procedures using the previously validated and/or published administrative codes.
4. Does the study limit its assessment to only in-hospital outcomes rather than those occurring after discharge? Yes
5. Does the study distinguish complications from comorbidities or clearly not where it cannot? Yes
Section C: Data Analysis
6. Does the study clearly account for the survey design of the NIS and its components - clustering, stratification, and weighting? Yes. All analyses were conducted accounting for survey nature (SURVEYMEANS, SURVEYLOGISTIC, and SURVEYFREQ), clustering (HOSP_NIS), weighting (DISCWT), and stratification (NIS_STRATUM) of the NIS.
7. Does the study adequately address changes in data structure over time (for trend analyses)? Yes, we have clearly addressed this in the methods section.

**Table 4:** ICD diagnostic codes used in the study ICD CM/PCS, international classification of diseases, clinical modification, and procedure codes

Diagnosis/Procedure	ICD CM/PCS codes
Primary sclerosing cholangitis	K83.01, 576.1
Endoscopic retrograde cholangiopancreatography	51.10, 51.11, 52.13, 0FJB8ZZ, 0FJD8ZZ, 0F9C8ZZ, 0F758DZ, 0F768DZ, 0F788DZ, 0F798DZ, 0F7C8DZ, 0F9580Z, 0F9680Z, 0F9880Z, 0F9980Z, 0F9C80Z, 0FC58ZZ, 0FC68ZZ, 0FC88ZZ, 0FC98ZZ, 0FCC8ZZ, 0FF58ZZ, 0FF68ZZ, 0FF88ZZ, 0FF98ZZ, 0FFC8ZZ, 0FJD8ZZ, 0F7D8DZ, 0F7F8DZ, 0F9D80Z, 0F9F80Z, 0FCD8ZZ, 0FCF8ZZ, 0FFD8ZZ, 0FFF8ZZ
Introduction of other anti-infective into peripheral Vein	99.21, 99.22, 3E03029
Post-ERCP cholangitis	K830, K8308
Post-ERCP pancreatitis	577.0, K91.89
Post-procedure hemorrhage	459.01, K9184, K91840, K91841
Post-ERCP septicemia	A40.0, A40.1, A40.2, A40.3, A40.8, A40.9, A41.0, A41.01, A41.02, A41.1, A41.2, A41.3, A41.4, A41.5, A41.51, A41.52, A41.53, A41.54, A41.59, A41.8, A41.81, A41.89, A41.9, R65.20, R65.21, R78.81
Cholecystitis	K81.0, K83.0
Peritonitis	K65.9
Pneumonia	J13, J15.9, J18.0, J18.8, J18.9, J85.2
Perforation	K22.3, K27.5, K63.1
